# Altered Pathogen Spectrum of Spontaneous Bacterial Peritonitis in Patients Treated With Proton Pump Inhibitors

**DOI:** 10.1111/apt.70593

**Published:** 2026-02-17

**Authors:** Philip Kitchen, Sarah L. Schütte, Tammo L. Tergast, Benjamin Maasoumy, Leonie Braun, Ulrich vor dem Esche, Georg Häcker, Marcus M. Mücke, Marlene Reincke, Michael Schultheiss, Robert Thimme, Siegbert Rieg, Dominik Bettinger, Lukas Sturm

**Affiliations:** ^1^ Department of Medicine II, Medical Center University of Freiburg, Faculty of Medicine University of Freiburg Freiburg Germany; ^2^ Department of Gastroenterology, Hepatology, Infectious Diseases and Endocrinology Hannover Medical School Hannover Germany; ^3^ German Center for Infection Research (DZIF) Hannover/Braunschweig Germany; ^4^ Institute for Medical Microbiology and Hygiene, Medical Center University of Freiburg, Faculty of Medicine University of Freiburg Freiburg Germany; ^5^ Department of Internal Medicine I, University Hospital Goethe‐University Frankfurt Frankfurt am Main Germany; ^6^ Berta‐Ottenstein‐Programme, Faculty of Medicine University of Freiburg Freiburg Germany

**Keywords:** cirrhosis, decompensation, infection

## Abstract

**Background:**

Proton pump inhibitor (PPI) treatment leads to significant changes in the gut microbiota composition. We hypothesized that these alterations may transfer into distinct changes in the causative agents of spontaneous bacterial peritonitis (SBP) in patients with cirrhosis.

**Aims:**

To investigate the impact of PPI treatment on the pathogen spectrum of SBP.

**Methods:**

A total of 227 cirrhosis patients with culture‐positive SBP from two German tertiary care centers were included and the pathogen spectrum of SBP was analysed with respect to PPI treatment.

**Results:**

In the PPI‐group, gram‐positive bacteria were significantly more common compared to the non‐PPI‐group (62.1% vs. 34.0%, *p* < 0.001). This involved increased abundances of *Enterococcus* spp. (22.0% vs. 8.0%), *Streptococcus* spp. (14.7% vs. 6.0%) and *Staphylococcus aureus* (15.8% vs. 8.0%). Importantly, more than half of the *Enterococcus* isolates were susceptible only to antibiotics with extended gram‐positive activity. Conversely, gram‐negative bacteria were significantly more frequent in the non‐PPI‐group compared to the PPI‐group (64.0% vs. 40.7%, *p* = 0.003), primarily due to a higher prevalence of *E. coli* (40.0% vs. 20.3%). In uni‐ and multivariable logistic regression analyses, PPI use was an independent predictor of gram‐positive SBP (OR 2.719, 95% CI 1.343–5.506, *p* = 0.005), adjusted for nosocomial infection (OR 2.609, 95% CI 1.464–4.650, *p* = 0.001) and Child‐Pugh score (OR 0.684, 0.552–0.848, *p* < 0.001).

**Conclusions:**

PPI treatment is associated with increased abundances of gram‐positive pathogens, including *Enterococcus* spp., in culture‐positive SBP. This result can help guide empiric antibiotic therapy of SBP.

## Graphical abstract

This multicenter, observational study reveals distinct alterations in the microbial spectrum of spontaneous bacterial peritonitis (SBP) associated with proton pump inhibitor use. These results are especially relevant for guiding empiric antibiotic therapy of SBP.

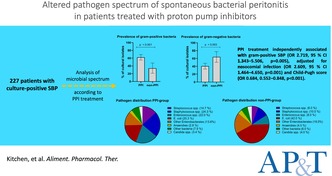

**Abbreviations**
CI: confidence interval
OR: odds ratio
PPI: proton pump inhibitor
SBP: spontaneous bacterial peritonitis

## 1 Introduction

Spontaneous bacterial peritonitis (SBP) is a serious infectious complication of advanced chronic liver disease with a reported prevalence of up to over 20% among patients hospitalised with decompensated cirrhosis [1]. The development of SBP has a significant prognostic impact, as the in‐hospital mortality rate of patients with SBP is more than 15% and one‐year mortality after SBP exceeds 50% [2]. Hence, timely diagnosis and adequate antibiotic treatment of SBP are critical. The pathogen spectrum of SBP is a crucial factor in this regard, as it may significantly affect the choice and efficacy of empiric antibiotic treatment. Therefore, identifying determinants of the pathogen spectrum of SBP is essential. Taking into consideration that SBP results from pathologic translocation of bacteria from the gut into ascites, effectors of the gut microbiota haemostasis may be particularly relevant in this context. A variety of previous studies have established the significant impact of treatment with proton pump inhibitors (PPI) on the gut microbiota composition [3, 4, 5]. Based on this evidence, we hypothesized that PPI treatment may be associated with distinct alterations of the causative agents of SBP. Therefore, the aim of the present study was to investigate the impact of PPI treatment on the pathogen spectrum of SBP in patients with cirrhosis.

## 2 Methods

### 2.1 Patient Selection and Data Collection

**FIGURE 1:**
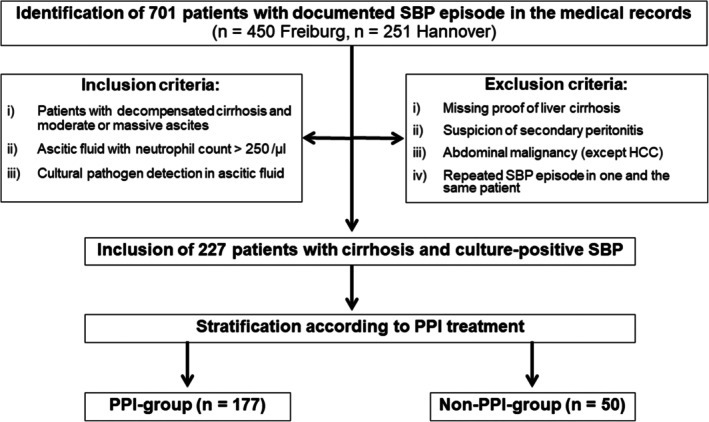
Patient inclusion. A total of 701 consecutive patients with documentation of an SBP episode were identified at two German tertiary care centers and screened for study eligibility. After application of inclusion and exclusion criteria, 227 patients with cirrhosis and culture‐positive SBP were included and stratified according to PPI treatment into a PPI‐group (*n* = 177) and a non‐PPI‐group (*n* = 50). HCC, hepatocellular carcinoma; PPI, proton pump inhibitor; SBP, spontaneous bacterial peritonitis.

Figure 1 summarises patient inclusion. A total of 701 patients with documentation of an episode of SBP between January 2007 and July 2025 were retrospectively identified in the medical records of two German tertiary care centers (*n* = 450 Medical Center University of Freiburg, *n* = 251 Hannover Medical School). An a priori study size calculation was not performed but all identified patients were screened for study eligibility. Inclusion criteria were (i) patients hospitalised with decompensated cirrhosis and moderate or massive ascites, (ii) ascitic fluid with neutrophil count of > 250/μL and (iii) cultural pathogen detection in the ascitic fluid. Exclusion criteria were (i) missing proof of liver cirrhosis, (ii) suspicion of secondary peritonitis, (iii) abdominal malignancies except for hepatocellular carcinoma and (iv) recurrent SBP in one and the same patient in the study collective (exclusion criteria are specified in Table S1). After application of these criteria, 227 cirrhosis patients with culture‐positive SBP were included in the study.

The pathogens detected in ascites culture were recorded. Further, the medical records were reviewed and clinical data of the included patients were extracted. Special focus was put on whether patients received PPI treatment at the time of SBP diagnosis, and patients were stratified into a PPI‐group (*n* = 177) and a non‐PPI‐group (*n* = 50). Type of PPI medication, daily dose, and changes in PPI treatment within 4 weeks prior to SBP diagnosis were assessed.

### 2.2 Ethics Statement

The study was approved by the local ethics committees (no. EK 21‐1074 and 3193‐2016) and is in accordance with the Declaration of Helsinki.

### 2.3 Statistical Analyses

Categorial variables were expressed as frequency and percentage, metric variables as median with interquartile range. The pathogen spectrum of SBP was analysed stratified according to PPI treatment. Differences between PPI‐ and non‐PPI group were determined using the Chi‐square test in case of categorical variables and the Mann–Whitney‐*U* test in case of metric variables (in the absence of a Gaussian distribution of the data). To identify predictors of the pathogen spectrum of SBP and to adjust for potential imbalances between PPI‐group and non‐PPI‐group, uni‐ and multivariable logistic regression analyses were performed (forward variable selection, likelihood ratio, *p* < 0.05 in; *p* > 0.10 out). *p*‐values < 0.05 were considered to be statistically significant. Statistical analyses were performed with STATA (Version 17.0, Stata Corp LLC., Texas, USA) and SPSS (Version 28.0, IBM, New York, USA).

## 3 Results

### 3.1 Patient Baseline Characteristics

Characteristics of the included patients at the time of SBP diagnosis are summarised in Table 1. No significant differences were observed between PPI‐ and non‐PPI‐group regarding age, gender distribution and aetiology of chronic liver disease, with alcohol‐related liver disease being the leading cause of cirrhosis. Patients in the non‐PPI‐group had laboratory parameters indicative of more severely impaired liver function, which was also reflected by a numerically only slightly, but statistically significantly higher Child‐Pugh score (11 (11–12) vs. 11 (10–11), *p* = 0.028) and Model for End‐Stage Liver Disease 3.0 score (MELD 3.0; 26 (23–32) vs. 25 (23–27), *p* = 0.035). SBP was classified as hospital‐acquired in a significant proportion of patients in both PPI‐group and non‐PPI group (60.5% vs. 48.0%, *p* = 0.116). Prior hospitalisation within 90 days before SBP onset was recorded more frequently in the PPI‐group (82.5% vs. 62.0%, *p* = 0.002). Further, the PPI‐group had a higher prevalence of previous SBP episodes (28.4% vs. 14.0%, *p* = 0.038). However, not all of these patients received antibiotic secondary prophylaxis for SBP (16.9% in the PPI‐group vs. 8.0% in the non‐PPI‐group, *p* = 0.117).

**TABLE 1:** Baseline characteristics of patients.

	All patients	Non‐PPI group	PPI‐group	*p*
	(*n* = 227)	(*n* = 50)	(*n* = 177)	
Age [years]	59 (52–67)	58 (51–69)	60 (52–67)	0.893
Gender				0.901
Female	56 (24.7)	12 (24.0)	44 (24.9)	
Male	171 (75.3)	38 (76.0)	133 (75.1)	
Aetiology				0.783
ALD	115 (50.7)	24 (48.0)	91 (51.4)	
HCV	42 (18.5)	11 (22.0)	42 (18.5)	
HBV	6 (2.6)	1 (2.0)	6 (2.6)	
MASLD	12 (5.3)	4 (8.0)	12 (5.3)	
Other/unknown	52 (22.9)	10 (20.0)	52 (22.9)	
Ascites				0.002
Moderate	15 (6.6)	8 (16.0)	7 (4.0)	
Massive	212 (93.4)	42 (84.0)	170 (96.0)	
Hepatic encephalopathy	66 (29.1)	13 (26.0)	53 (29.9)	0.839
Grade				
I/II	49 (74.2)	10 (76.9)	39 (73.6)	
III/IV	17 (25.8)	3 (23.1)	14 (26.4)	
Variceal bleeding	19 (8.4)	7 (14.0)	12 (6.8)	0.104
Hepatocellular carcinoma	32 (14.1)	6 (12.0)	26 (14.7)	0.629
TIPS	34 (15.0)	5 (10.0)	29 (16.4)	0.264
Prior hospitalisation within last 90 days	177 (78.0)	31 (62.0)	146 (82.5)	0.002
History of SBP	57 (25.2)	7 (14.0)	50 (28.4)	0.038
SBP prophylaxis	34 (15.0)	4 (8.0)	30 (16.9)	0.117
Antibiotic				
Norfloxacin	16 (47.1)	1 (25.0)	15 (50.0)	
Ciprofloxacin	1 (2.9)	0	1 (3.3)	
Rifaximin	15 (44.1)	3 (75.0)	12 (40.0)	
Cotrimoxazol	2 (5.9)	0	2 (6.7)	
Recent systemic antibiotic treatment	60 (26.4)	13 (26.0)	47 (26.6)	0.938
Nosocomial SBP	131 (57.7)	24 (48.0)	107 (60.5)	0.116
ICU treatment	36 (15.9)	12 (24.0)	24 (13.6)	0.074
Bacteremia	39 (22.3)	8 (22.9)	31 (22.1)	0.928
Child‐pugh stage				
A	1 (0.4)	1 (2.0)	0	0.054
B	45 (19.8)	6 (12.0)	45 (19.8)	
C	181 (79.7)	43 (86.0)	181 (79.7)	
Child‐pugh score	11 (10–11)	11 (11–12)	11 (10–11)	0.028
MELD score	20 (15–26)	22 (18–30)	20 (15–25)	0.089
MELD 3.0 score	25 (23–29)	26 (23–32)	25 (23–27)	0.035
Laboratory parameters				
White blood cells [10^3^/μL]	9.9 (6.3–14.2)	10.5 (5.4–14.8)	9.9 (6.4–14.1)	0.875
Platelets [10^3^/μL]	104 (64–174)	116 (76–192)	99 (63–164)	0.187
Creatinine [mg/dL]	1.6 (1.1–2.5)	1.6 (1.1–2.7)	1.6 (1.2–2.5)	0.957
INR	1.5 (1.3–1.7)	1.5 (1.4–1.8)	1.4 (1.3–1.7)	0.063
Bilirubin [mg/dL]	3.3 (1.6–7.8)	5.3 (2.5–10.3)	3.0 (1.4–6.5)	0.02
Albumin [g/dL]	2.5 (2.1–2.8)	2.4 (2.1–2.6)	2.6 (2.1–2.9)	0.063
AST [U/L]	61 (39–98)	86 (54–174)	58 (38–85)	0.002
ALT [U/L]	34 (20–56)	37 (22–75)	33 (19–53)	0.112
Sodium [mmol/L]	133 (128–138)	133 (128–136)	134 (128–138)	0.508
C‐reactive protein [mg/L]	69 (30–122)	50 (29–110)	74 (30–123)	0.272
Procalcitonin [ng/mL]	69 (30–122)	2.6 (1.7–5.7)	2.4 (0.6–7.8)	0.654
Neutrophiles ascites [10^3^/μL]	2.9 (1.1–6.5)	3.9 (1.4–8.1)	2.7 (0.9–5.9)	0.034
PPI medication				
Pantoprazole			171 (96.6)	
Esomeprazole			5 (2.8)	
Omeprazole			1 (0.6)	
PPI daily dose [mg]^a^				
20 mg			16 (9.0)	
40 mg			102 (57.6)	
80 mg			59 (33.3)	

*Note:* Data are presented as number (percentage) and median (interquartile range).

Abbreviations: ALD, alcohol‐related liver disease; ALT, alanine aminotransferase; AST, aspartate aminotransferase; HBV/HCV, hepatitis B/C virus; ICU, intensive care unit; INR, international normalised ratio; MASLD, metabolic dysfunction‐associated liver disease; MELD, Model for End‐Stage Liver Disease; PPI, proton pump inhibitor; SBP, spontaneous bacterial peritonitis; TIPS, transjugular intrahepatic portosystemic shunt.

^a^ Pantoprazole dose equivalent, i.e., dose of omeprazole and esomeprazole equals double the pantoprazole dose.

### 3.2 Increased Gram‐Positive SBP in PPI‐Treated Patients

**FIGURE 2:**
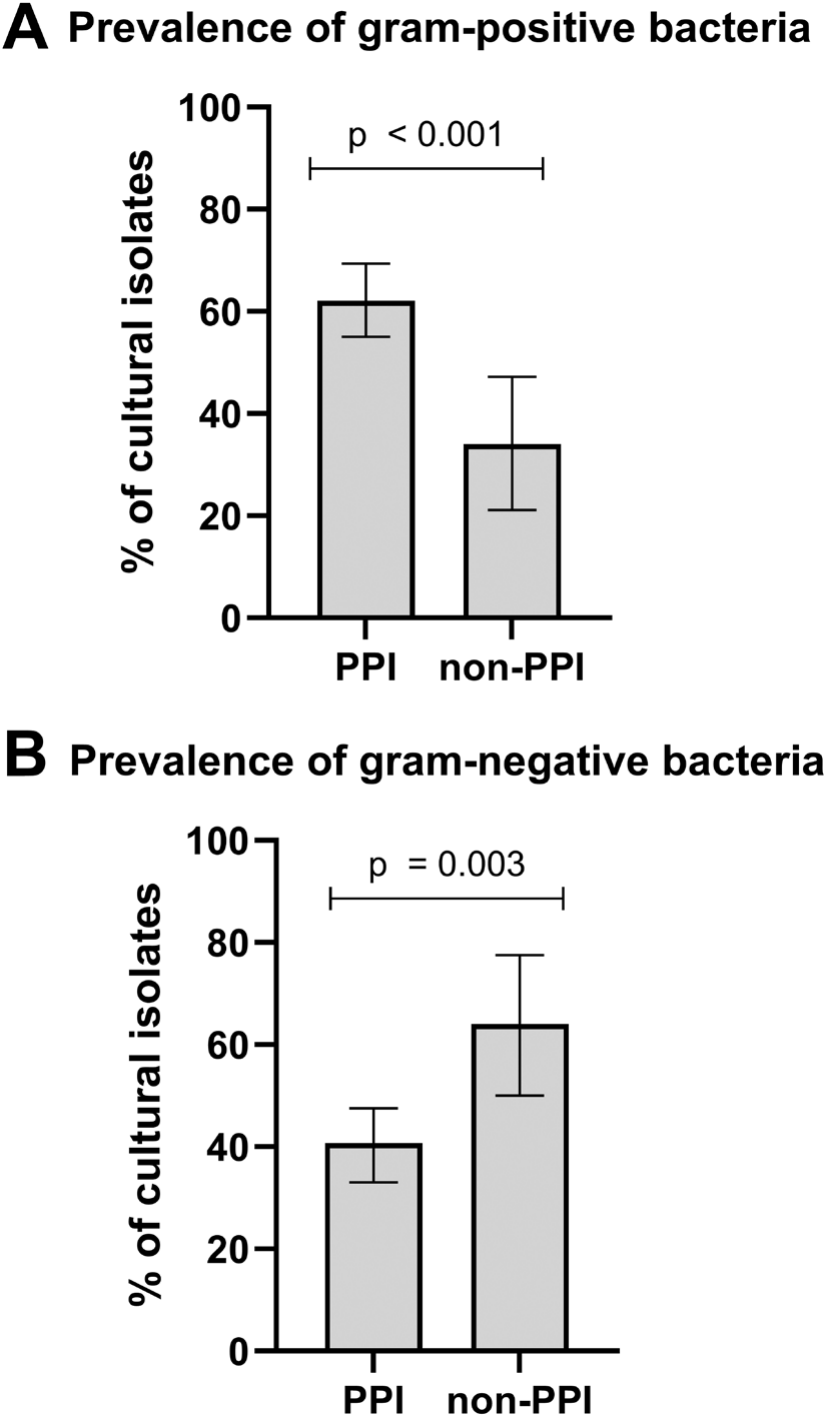
Gram‐status of bacterial isolates in PPI group and non‐PPI group. PPI treatment was strongly linked to the gram‐status of the pathogens of SBP: Gram‐positive SBP was significantly more frequent in the PPI group (62.1% vs. 34.0%, *p* < 0.001; panel A), while gram‐negative SBP was significantly more common in the non‐PPI group (64.0% vs. 40.7%, *p* = 0.003; panel B). Error bars indicate 95% confidence interval. PPI, proton pump inhibitor; SBP, spontaneous bacterial peritonitis.

As to be expected, a great majority of 93.4% of patients had mono‐microbial infection, while two or three different pathogens were identified in 4.8% and 1.8% of patients, respectively. In the PPI‐group, gram‐positive bacteria were isolated significantly more frequently in comparison to the non‐PPI‐group (62.1% vs. 34.0%, *p* < 0.001; Figure 2A). Conversely, gram‐negative bacteria were identified significantly more often in the non‐PPI‐group compared to the PPI‐group (64.0% vs. 40.7%, *p* = 0.003; Figure 2B).

The increased frequency of gram‐positive infection in the PPI‐group compared with the non‐PPI‐group was particularly evident in cases of nosocomial SBP (71.0% vs. 41.7%, *p* = 0.006). In community‐acquired SBP, the proportion of gram‐positive infections was also markedly higher in the PPI‐group, although the difference did not reach statistical significance (48.6% vs. 26.9%, *p* = 0.057). The increased frequency of gram‐positive SBP in the PPI‐group was consistent in sub‐analyses excluding patients receiving SBP prophylaxis (62.6% vs. 32.6%, *p* < 0.001) and patients with recent systemic antibiotic treatment (63.1% vs. 35.1%, *p* = 0.002). Further, the higher rate of gram‐positive SBP in the PPI‐group was also consistent when excluding patients (*n* = 22) in whom PPI treatment had been started or discontinued within the last 30 days prior to SBP development (60.8% vs. 29.8%, *p* < 0.001). Analyses stratified by the time point of SBP within the study inclusion period are summarised in Figure S1.

### 3.3 Pathogen Spectrum of SBP on Genus and Species Level

**FIGURE 3:**
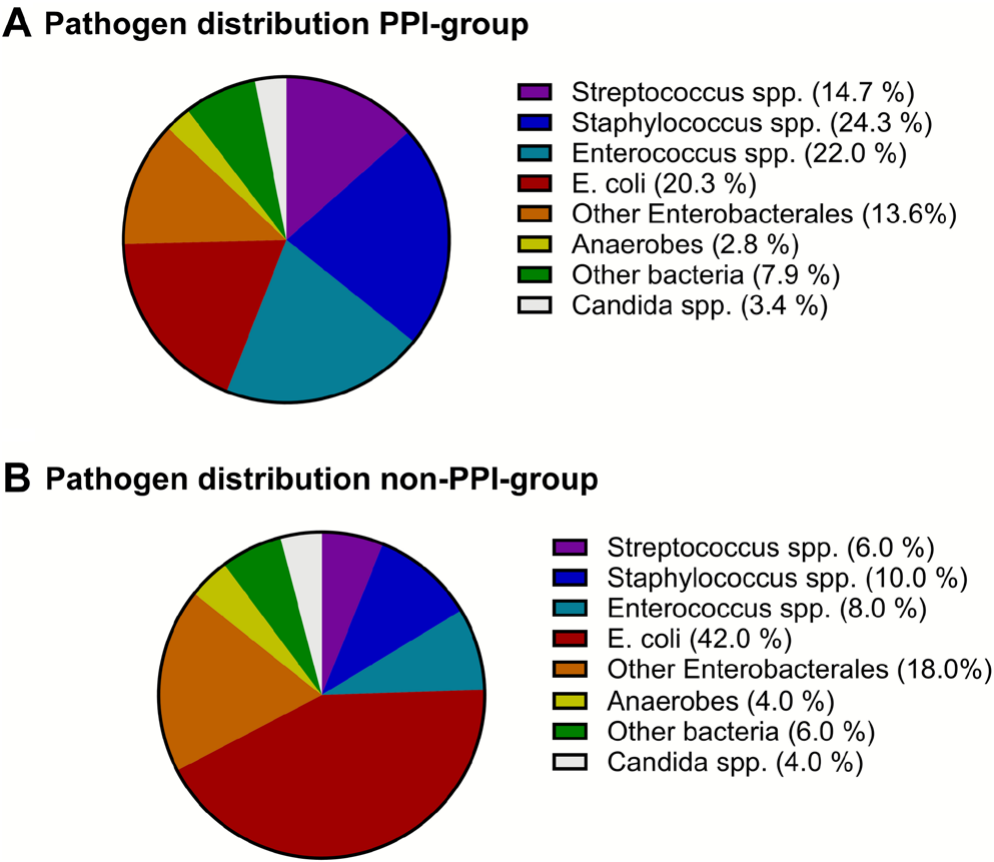
Cultural isolates in PPI‐group and non‐PPI‐group. SBP was caused by *Enterococcus* spp., *Streptococcus* spp., and *Staphylococcus* spp. more commonly in the PPI‐group (panel A), while SBP was predominantly caused by *E. coli* and other gram‐negative *Enterobacterales* in the non‐PPI‐group (panel B). PPI, proton pump inhibitor; SBP, spontaneous bacterial peritonitis.

Analyses of the cultural pathogen spectrum of SBP on genus and species level showed that *Enterococcus* spp. were almost three‐fold more common in the PPI‐group compared to the non‐PPI‐group (22.0% vs. 8.0%; Figure 3A). Of note, species typically susceptible only to antibiotics with extended gram‐positive activity, such as *E. faecium* , accounted for more than half of the *Enterococcus* isolates (60.5% in the PPI‐group, 75.0% in the non‐PPI‐group). Further, *Streptococcus* spp. (14.7% vs. 6.0%) and *Staphylococcus* spp., including *S. aureus* (15.8% vs. 8.0%) as well as *coagulase‐negative Staphylococci* (8.5% vs. 2.0%) were significantly more common in the PPI‐group. In turn, the higher prevalence of gram‐negative SBP in the non‐PPI‐group was mainly due to increased abundances of *E. coli* (42.0% vs. 20.3%; Figure 3B). A detailed summary of the pathogen distribution for both groups is provided in Table S2. Analyses of microbial resistance patterns of the cultural isolates in the study collective showed that the overall prevalence of multi‐resistant bacteria was comparable in PPI‐group and non‐PPI‐group (10.7% vs. 10.0% of isolates, *p* = 0.881; Table S3).

### 3.4 Predictors of the Pathogen Spectrum of SBP

To identify clinically relevant predictors of the pathogen spectrum of SBP, uni‐ and multivariable regression analyses were performed. These showed that PPI use independently increased the odds for gram‐positive SBP by more than the 2.5‐fold (OR 2.719, 95% CI 1.343–5.506, *p* = 0.005; Table 2). In this context, the daily dose of PPI medication was not significantly associated with the odds of gram‐positive infection. Besides PPI treatment, nosocomial infection (OR 2.609, 95% CI 1.464–4.650, *p* = 0.001) and the Child‐Pugh score (OR 0.684, 0.552–0.848, *p* < 0.001) emerged as independent predictors of gram‐positive SBP. PPI treatment also independently predicted gram‐positive SBP in a second, alternative regression model (Table 2).

**TABLE 2:** Predictors of gram‐positive SBP.

Parameters	Model 1					
	Univariable regression			Multivariable regression		
	OR	95% CI	*p*	OR	95% CI	*p*
Age [years]	1.010	0.988–1.031	0.375			
Female gender	0.771	0.417–1.427	0.408			
Alcohol‐related liver disease	1.210	0.716–2.044	0.477			
Antibiotic SBP prophylaxis	1.148	0.548–2.406	0.714			
Prior systemic antibiotic treatment	0.866	0.479–1.566	0.635			
Prior hospitalisation	1.106	0.589–2.078	0.754			
Nosocomial SBP	2.564	1.491–4.407	< 0.001	2.609	1.464–4.650	0.001
Child‐Pugh score	0.681	0.555–0.836	< 0.001	0.684	0.552–0.848	< 0.001
PPI treatment	3.187	1.648–6.162	< 0.001	2.719	1.343–5.506	0.005
PPI daily dose [mg]	1.005	0.990–1.020	0.494			

	Model 2					
	Univariable regression			Univariable regression		
	OR	95% CI	*p*	OR	95% CI	*p*
Nosocomial SBP	2.564	1.491–4.407	< 0.001	2.437	1.399–4.247	0.002
MELD 3.0	0.963	0.922–1.006	0.089			
PPI treatment	3.187	1.648–6.162	< 0.001	3.006	1.532–5.899	0.001

Abbreviations: CI, confidence interval; MELD, Model for End‐Stage Liver Disease; OR, odds ratio; PPI, proton pump inhibitor; SBP, spontaneous bacterial peritonitis.

### 3.5 Validation in an Independent Cohort

To validate the main results, cases of culture‐positive SBP (*n* = 24 PPI, *n* = 6 non‐PPI) were identified among an independent study collective of cirrhosis patients with bacterial infection published previously [6]. Despite the limited number of cases, analyses of the microbial spectrum indicated an association of PPI treatment with the pathogens of SBP comparable to the findings in the primary study collective: While gram‐positive bacteria were isolated in a majority of 54.2% of PPI patients, only a single non‐PPI patient (16.7%) had gram‐positive SBP. *Enterococcus* spp. and *Staphylococcus* spp. were the most common gram‐positive pathogens among PPI patients (Table S4).

## 4 Discussion

In recent years, a number of studies have associated PPI treatment with an increased risk of complications in patients with cirrhosis. This includes the development of bacterial infections and of SBP in particular [7, 8]. Importantly, prior studies mainly focused on associations between PPI treatment and the incidence of SBP per se. However, PPI treatment may also have a critical impact on the characteristics of the infection. In this context, the microbial profile of SBP is an aspect with high clinical relevance. Indeed, the present study shows that in culture‐positive SBP PPI treatment is linked to distinct alterations of the pathogen spectrum. In particular, this involves a significantly higher rate of gram‐positive pathogens. For medical practice, this may be especially meaningful with regard to an almost threefold higher rate of SBP associated with *Enterococcus spp* in PPI‐treated patients. First, this genus of bacteria exhibits intrinsic resistance to cephalosporin antibiotics. This is important, since third generation cephalosporins are the recommended empiric antibiotic treatment for community‐acquired SBP [9, 10] (25.6% of SBP cases due to *Enterococcus* spp. were community‐acquired in the present study). Second, an increased probability of SBP due to *Enterococci* is also relevant for the management of nosocomial infections: Species such as *E. faecium* (over 50% of *Enterococcus* isolates in the present study) are mostly not susceptible to acylaminopenicillin/beta‐lactamase inhibitor or carbapenem antibiotics which are the first‐line treatment of nosocomial SBP, but require administration of substances with extended gram‐positive activity, such as daptomycin, vancomycin or linezolid [11, 12]. International guidelines recommend to make the decision for or against use of these antibiotics for empiric antibiotic treatment of SBP on the basis of the expected pathogen/microbial resistance spectrum [9, 10]. The present results may provide guidance for clinical decision making in this context. Besides SBP caused by *Enterococci*, the increased rate of *Staphylococcus* spp. in the PPI‐group may also be of prognostic relevance, as SBP due to *S. aureus* has been associated with significantly worse prognosis [13]. Further, coagulase‐negative *Staphylococci* often also require antibiotics with extended gram‐positive activity.

It is interesting to note that one previous study reported an increased mortality of SBP among patients with PPI treatment compared to patients who did not receive PPIs [14]. In this context, alterations of the pathogen spectrum and possibly inefficient empiric antibiotic therapy are factors that may contribute to an unfavourable outcome of PPI‐treated patients. Of course, this assumption remains speculative. In any case, the present results argue for awareness of an increased probability of gram‐positive pathogens including *Enterococci* and *Staphylococci* in patients with SBP who receive PPI treatment and to consider this factor when initiating empiric antibiotic therapy. The frequent prescription of PPIs in patients with cirrhosis in everyday medical practice further underscores the clinical relevance of this aspect [15]. Besides PPI treatment, Child‐Pugh score and nosocomial infection could be identified as meaningful predictors of the pathogen spectrum of SBP in our study. These results are in conformity with previous studies that have reported increased gram‐positive infections in nosocomial SBP and a decrease of gram‐positive pathogens with higher Child‐Pugh score [13, 16].

PPI treatment has a significant impact on the intestinal microbiota composition. A variety of studies have shown that PPI intake can lead to gut dysbiosis and small intestinal bacterial overgrowth. These factors may be particularly relevant in patients with advanced cirrhosis, as they are inherently prone to develop intestinal dysbiosis in the course of the disease [17, 18]. Distinct changes in the gut microbiota composition under PPI treatment have been described. These involve increased abundances of *Enterococci*, *Staphylococci*, and *Streptococci* in particular [3, 4, 5]. Considering the analogous shifts in the pathogen spectrum of SBP among PPI‐treated patients observed in the present study, it seems plausible to assume that these could reflect aberrations of the gut microbiota composition. This assumption is also supported by recent results that indicate increased gram‐positive bacterial translocation into the bloodstream in patients with cirrhosis who receive PPI treatment [19]. However, as analyses of the faecal microbiota composition were not available in our study collective, a direct connection between altered gut microbiota composition and the pathogen spectrum of SBP in PPI‐treated patients cannot be established on the basis of the present results.

Our study has some limitations that need to be addressed. First, we investigated cases of culture‐positive SBP. Naturally, this implies that effects of PPI treatment on the pathogen spectrum of SBP observed in the present study may not be fully transferable to culture‐negative infection. This may be a clinically relevant issue, as cultural pathogen detection is not successful in the majority of SBP cases [9]. In this context, next‐generation sequencing constitutes a promising approach to further investigate the pathogen spectrum of SBP and its effectors in future studies [20]. Another critical aspect of this study is selection bias with respect to PPI treatment. Multivariable regression analyses showed that PPI treatment indeed was an independent predictor of gram‐positive SBP in the study collective. However, bias introduced by confounders that were not adjusted for due to unavailability of data, such as dietary habits, cannot be fully excluded, of course. Eventually, from a clinical perspective, PPI treatment may be considered a relevant indicator of an altered microbial spectrum in patients with SBP independently of whether the underlying connection is merely associative or indeed causative. Naturally, our results require validation in further studies.

In conclusion, the present study shows that PPI treatment is associated with a significantly higher prevalence of gram‐positive infection in patients with cirrhosis and culture‐positive SBP, including increased abundances of *Enterococcus* spp. and *Staphylococcus* spp. These results may provide guidance for clinical decision making, especially with respect to empiric antibiotic therapy in patients with SBP.

## Author Contributions

**Philip Kitchen:** conceptualization, methodology, investigation, formal analysis, data curation, writing – original draft. **Sarah L. Schütte:** investigation, data curation. **Tammo L. Tergast:** investigation, data curation. **Benjamin Maasoumy:** writing – review and editing. **Leonie Braun:** investigation, data curation. **Ulrich vor dem Esche:** investigation, data curation. **Georg Häcker:** writing – review and editing. **Marcus M. Mücke:** investigation, data curation. **Marlene Reincke:** writing – review and editing. **Michael Schultheiss:** writing – review and editing. **Robert Thimme:** writing – review and editing. **Siegbert Rieg:** writing – review and editing. **Dominik Bettinger:** writing – review and editing. **Lukas Sturm:** conceptualization, methodology, formal analysis, writing – original draft, supervision.

## Funding

The authors have nothing to report.

## Ethics Statement

The study was approved by the local ethics committees (no. EK 21‐1074 and 3193‐2016) and is in accordance with the Declaration of Helsinki.

## Consent

Due to the retrospective nature of the study, informed patient consent was waived.

## Conflicts of Interest

The authors declare no conflicts of interest.

## Supporting information


**Data S1:** Supporting Information.

## Data Availability

The data that support the findings of this study are available on request from the corresponding author. The data are not publicly available due to privacy or ethical restrictions.
