# Supplementary material for: Altered Pathogen Spectrum of Spontaneous Bacterial Peritonitis in Patients Treated With Proton Pump Inhibitors
Source: Aliment Pharmacol Ther. 2026 Feb 17;63(11):1487–94. doi: 10.1111/apt.70593 (PMC13170620; doi:10.1111/apt.70593)
Supplement: Supplementary file 1 — Data S1: Supporting Information. [file APT-63-1487-s001.docx]

**Supplementary files: Altered pathogen spectrum of spontaneous bacterial peritonitis in patients treated with proton pump inhibitors**

Philip Kitchen, Sarah L. Schütte, Tammo L. Tergast, Benjamin Maasoumy, Leonie Braun, Ulrich vor dem Esche, Georg Häcker, Marcus M. Mücke, Marlene Reincke, Michael Schultheiss, Robert Thimme, Siegbert Rieg, Dominik Bettinger and Lukas Sturm

**Contents**

Supplementary table 1 – page 2

Supplementary table 2 – page 3

Supplementary table 3 – page 5

Supplementary table 4 – page 6

Supplementary figure 1 – page 7

**Supplementary table 1: Exclusion criteria**

| **Exclusion criteria** | **Specification** |
| --- | --- |
| Missing proof of liver cirrhosis | No clear evidence for cirrhosis on the basis of  i) pathognomonic clinical, laboratory and sonographic/elastographic findings  and/or  ii) liver biopsy |
| Suspicion of secondary peritonitis | Recent abdominal surgery  Gastrointestinal perforation  Gastrointestinal ischemia  Acute pancreatitis  Severe gastrointestinal inflammation (e. g. florid ulcerative colitis)  Recent severe abdominal trauma |
| Abdominal malignancies | Gastrointestinal adenocarcinomas  Pancreatic and biliary cancers  Ovarian and uterine malignancies  Neuroendocrine tumors  Lymphoma  Primary or metastatic peritoneal tumors  Hepatocellular carcinoma on the grounds of liver cirrhosis without proof of peritoneal carcinomatosis was not considered an exclusion criterium |

**Supplementary table 2: Cultural pathogen spectrum of SBP**

|  | **All patients**  **(n = 227)** | **Non-PPI group**  **(n = 50)** | **PPI-group**  **(n = 177)** |
| --- | --- | --- | --- |
| **Streptococcus spp.**  S. adiacens  S. agalacticae  S. anginosus  S. gallolyticus  S. gordonii  S. mitis  S. parasanguinis  S. pneumonia  S. pyogenes  S. salivarius  S. sanguinis  S. vestibularis | 29 (12.8)  1 (0.4)  2 (0.9)  8 (3.5)  1 (0.4)  2 (0.9)  4 (1.8)  3 (1.3)  2 (0.9)  1 (0.4)  2 (0.9)  1 (0.4)  2 (0.9) | 3 (6.0)  0  1 (2.0)  1 (2.0)  0  0  0  0  0  1 (2.0)  0  0  0 | 26 (14.7)  1 (0.6)  1 (0.6)  7 (4.0)  1 (0.6)  2 (1.1)  4 (2.3)  3 (1.7)  2 (1.1)  0  2 (1.1)  1 (0.6)  2 (1.1) |
| **Staphylococcus spp.**  S. aureus  S. auricularis  S. epidermidis  S. haemolyticus  S. hominis  S. saprophyticus | 48 (21.1)  32 (14.1)  2 (0.9)  8 (3.5)  4 (1.8)  1 (0.4)  1 (0.4) | 5 (10.0)  4 (8.0)  1 (2.0)  0  0  0  0 | 43 (24.3)  28 (15.8)  1 (0.6)  8 (4.5)  4 (2.3)  1 (0.6)  1 (0.6) |
| **Enterococcus spp.**  E. faecalis  E. faecium  E. casseliflavus  E. cecorum  E. gallinarum  E. mallodratus | 43 (18.9)  17 (7.5)  21 (9.3)  1 (0.4)  1 (0.4)  2 (0.9)  1 (0.4) | 4 (8.0)  1 (2.0)  2 (4.0)  0  0  1 (2.0)  0 | 39 (22.0)  16 (9.0)  19 (10.7)  1 (0.6)  1 (0.6)  1 (0.6)  1 (0.6) |
| **E. coli** | 57 (25.1) | 21 (42.0) | 36 (20.3) |
| **Klebsiella spp.**  K. ornitholytica  K. oxytoca  K. pneumoniae  K. variicola | 22 (9.7)  1 (0.4)  5 (2.2)  15 (6.6)  1 (0.4) | 5 (10.0)  0  2 (4.0)  3 (6.0)  0 | 17 (9.6)  1 (0.6)  3 (1.7)  12 (6.7)  1 (0.6) |
| **Other Enterobacterales**  Citrobacter koseri  Enterobacter cancerogenus  Enterobacter cloacae  Providencia rettgeri  Salmonella typhimurium  Serratia marcescens | 11 (4.8)  1 (0.4)  1 (0.4)  5 (2.2)  1 (0.4)  1 (0.4)  2 (0.9) | 4 (8.0)  0  1 (2.0)  1 (2.0)  1 (2.0)  1 (2.0)  0 | 7 (4.0)  1 (0.6)  0  4 (2.3)  0  0  2 (1.1) |
| **Anaerobic bacteria**  Bacteroides fragilis  Clostridium spp.  Fusobacterium nucleatum  Parabacteroides distasonis  Prevotella oralis  Prevotella nigrescens | 7 (3.1)  2 (0.9)  1 (0.4)  1 (0.4)  1 (0.4)  1 (0.4)  1 (0.4) | 2 (4.0)  1 (2.0)  1 (2.0)  0  0  0  0 | 5 (2.8)  1 (0.6)  0  1 (0.6)  1 (0.6)  1 (0.6)  1 (0.6) |
| **Other bacteria**  Acinetobacter baumanii  Acinetobacter Iwofii  Bacillus spp.  Corynebacterium amycolatum  Corynebacterium jeikeium  Corynebacterium minutissium  Listeria monocytogenes  Micrococcus spp.  Pseudomonas aeruginosa  Prevotella oralis  Stenotrophomonas maltophilia | 17 (7.5)  4 (1.8)  1 (0.4)  1 (0.4)  2 (0.9)  1 (0.4)  1 (0.4)  2 (0.9)  1 (0.4)  2 (0.9)  1 (0.4)  1 (0.4) | 3 (6.0)  0  0  0  1 (2.0)  0  1  0  0  2 (4.0)  0  0 | 14 (7.9)  4 (2.3)  1 (0.6)  1 (0.6)  1 (0.6)  1 (0.6)  0  2 (1.1)  1 (0.6)  0  1 (0.6)  1 (0.6) |
| **Candida**  C. albicans  C. glabrata | 8 (3.5)  6 (2.6)  2 (0.9) | 2 (4.0)  2 (4.0)  0 | 6 (3.4)  4 (2.3)  2 (1.1) |

Data are presented as number (percentage).

Abbreviations: PPI – proton pump inhibitor treatment, SBP – spontaneous bacterial peritonitis

**Supplementary table 3: Prevalence of multi-resistant bacteria**

|  | Total | Non-PPI group | PPI-group |
| --- | --- | --- | --- |
| VRE^a^ | 2 (4.7) | 0 | 2 (5.1) |
| MRSA^b^ | 3 (9.4) | 1 (25.0) | 2 (7.1) |
| 3MRGN^c^ | 15 (14.4) | 3 (9.4) | 12 (16.7) |
| 4MRGN^c^ | 2 (1.9) | 1 (3.1) | 1 (1.4) |
| ESBL^c^ | 2 (1.9) | 2 (2.8) | 0 |

^a^ reported as number (percent of *Enterococcus spp.* isolates)

^b^ reported as number (percent of *Staphylococcus aureus* isolates)

^c^ reported as number (percent of gram-negative isolates)

Abbreviations: ESBL – extended spectrum beta-lactamase, MRGN – multiresistant gram-negative (bacteria), MRSA – methicillin-resistant Staphylococcus aureus, VRE – vancomycin-resistant Enterococcus

**Supplementary table 4: Cultural pathogen spectrum of SBP in validation cohort**

|  | **All patients**  **(n = 30)** | **Non-PPI**  **(n = 6)** | **PPI**  **(n = 24)** |
| --- | --- | --- | --- |
| **Gram-positive**  *Enterococcus spp.*  *Coagulase-neg. Staphylococci*  *Staphylococcus aures*  *Streptococcus spp.* | 14 (46.7)  5 (16.7)  6 (20.0)  2 (6.7)  1 (3.3) | 1 (16.7)  1 (16.7)  0  0  0 | 13 (54.2)  4 (16.7)  6 (25.0)  2 (8.3)  1 (4.2) |
| **Gram-negative**  *Escherichia coli*  *Other* | 16 (53.3)  13 (43.3)  3 (10.0) | 5 (83.3)  5 (83.3)  0 | 11 (45.8)  8 (33.3)  3 (12.5) |

Data are presented as number (percentage).

Abbreviations: PPI – proton pump inhibitor treatment, SBP – spontaneous bacterial peritonitis

**Supplementary figure 1: Gram-status of cultural isolates stratified by time point of SBP within the study inclusion period**


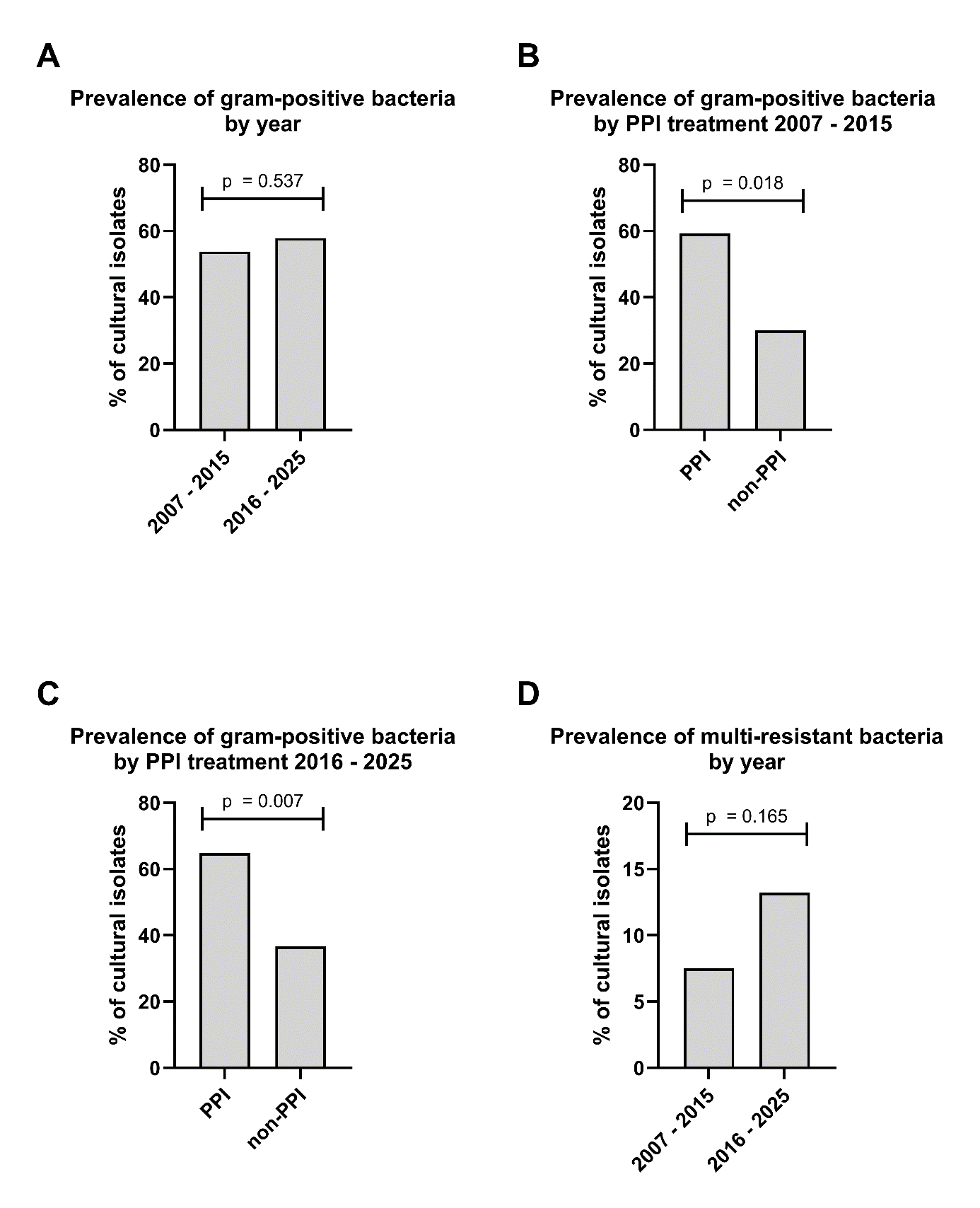


Distinct analyses of SBP cases from 2007 to 2015 (n = 106, 46.8 % of included patients) and cases from 2016 to 2025 (n = 121, 53.2 % of included patients) showed a comparable prevalence of gram-positive SBP cases in both time periods (53.8 % vs. 57.9 %; panel A). The increased frequency of gram-positive SBP in the PPI-group compared to the non-PPI-group was consistent in both time periods (59.3 % vs. 30.0 %, p = 0.018 and 64.8 % vs. 36.7 %, p = 0.007; panel B + C). An increase of multiresistant pathogens during the study period was observable (2007 – 2015: 7.5 %, 2016 – 2025: 13.2 %; panel D).

Abbreviations: PPI – proton pump inhibitor, SBP – spontaneous bacterial peritonitis
